# Genetic and Environmental Variation in Starch Content, Starch Granule Distribution and Starch Polymer Molecular Characteristics of French Bread Wheat

**DOI:** 10.3390/foods10020205

**Published:** 2021-01-20

**Authors:** Larbi Rhazi, Benoît Méléard, Olfa Daaloul, Guénolé Grignon, Gérard Branlard, Thierry Aussenac

**Affiliations:** 1 Institut Polytechnique UniLaSalle, Université d’Artois, ULR 7519, 19 rue Pierre Waguet, BP 30313, 60026 Beauvais, France; larbi.rhazi@unilasalle.fr; 2 ARVALIS—Institut du Végétal, F-91720 Boigneville, France; B.MELEARD@arvalis.fr (B.M.); guenole.grignon@axereal.com (G.G.); 3 National Agronomic Institute of Tunisia, University of Carthage, 43 Avenue Charles Nicolle, Tunis Mahrajène 1082, Tunisia; olfadaaloul.inat.iaa@gmail.com; 4 INRAE, UCA UMR1095 GDEC, 5 Chemin de Beaulieu, 63100 Clermont-Ferrand, France; gerard.branlard@gmail.com

**Keywords:** wheat, starch, MWD of starch polymers, environment and genetic impact

## Abstract

This study investigates genetic and environmental variation in starch content and characteristics of 14 French bread cultivars. Understanding the impact of these factors on wheat quality is important for processors and especially bakers to maintain and meet the requirements of industrial specifications. Different traits were evaluated: starch content, distribution of starch granules, percentage of amylose and amylopectin and their molecular characteristics (weight-average molar mass, number-average molar mass, polydispersity and gyration radius). Genetic, environment and their interaction had significant effects on all parameters. The relative magnitude of variance attributed to growth conditions, for most traits, was substantially higher (21% to 95%) than that attributed to either genotype (2% to 73%) or G × E interaction (2% to 17%). The largest environmental contribution (95%) to total variance was found for starch dispersity. The highest genetic influence was found for the percentage of A-type starch granules. G × E interaction had relatively little influence (≈7%) on total phenotypic variance. All molecular characteristics were much more influenced by environment than the respective percentages of amylose and amylopectin were. This huge difference in variance between factors obviously revealed the importance of the effect of growing conditions on characteristics of cultivars.

## 1. Introduction

Wheat flour composition and quality are tightly related to wheat kernel development and storage molecule accumulation which are strongly influenced by genetic and environmental factors [1,2,3]. Growing conditions presenting abiotic stresses such as elevated temperature, water deficit and drought stresses, have a considerable effect on wheat grain filling, yield and quality by impacting both nitrogen and carbon metabolism [4]. Changes in the accumulation of the major grain components are known to affect beadmaking quality. Heat stress during the grain filling period has been demonstrated to be one of the factors most affecting dough characteristics and wheat quality properties. High temperatures during grain development decrease grain size and thousand kernel weight, while increased temperatures before anthesis reduce the grain number in wheat [5]. Over the last 15 years, we have observed strong variations in the breadmaking quality of French wheat due to an increase in the frequency of abiotic stresses between crop seasons. These quality variations are also amplified by intra-year variations characterized by occasional stress events (e.g., heat and drought stresses). As a result of the crucial role of wheat protein in the establishment of technological properties, most of the cereal science studies conducted to understand and determine the effect of genetic and environmental factors on grain quality have focused on these specific fractions [6]. The molecular features of starch, the major component of the wheat grain, usually corresponding to 68–72% (*w*/*w*) of grain dry weight, have been poorly studied.

Starch is found in the form of starch granules trapped in a protein matrix. Three classes of granule can be distinguished: type A granules with a diameter greater than 15 µm and a lenticular shape, type B granules between 5 and 15 µm with a spherical shape and type C granules which are smaller than 5 µm [7,8]. For practical purposes, C-type granules are often considered a subpopulation of the B-type granule fraction, as they represent only a minor portion of the total starch by weight. Starch granules are composed of 18% to 35% amylose (AML), which is a linear molecule consisting of α-(1,4)-linked *D*-glucopyranosyl units with a degree of polymerization (DP) in the range 500–6000 glucose residues. A fraction of the AML molecules are slightly branched by α-(1,6)-linkages [9,10]. The major component of typical wheat starch is amylopectin (AMP) (65–82%). It is composed of glycosyl monomers of which 5% are joined with α-1,6 linkages [11,12,13] and is a very large, highly branched chain molecule with a DP ranging from 3 × 10^5^ to 3 × 10^6^ glucose units. The formation of AML is mainly due to the granule bound starch synthase (GBSS) coded by the waxy genes [14,15] named Wx-A1, Wx-B1 and Wx-D1 [16,17]. GBSS catalyzes elongation of the AML molecule using ADP-glucose. These enzymes are coded by three genes, located on the short arm of chromosome 7A, the long arm of chromosome 4A and the short arm of chromosome 7D [18,19]. AMP is synthesized by the coordinated action of a set of isoenzymes: starch branching enzymes (SBE), starch synthases (SS) and starch debranching enzymes (SDB) [20,21,22]. The molecular structure, some physicochemical properties and end-use quality of starch from normal, partial waxy and waxy wheat have been exhaustively studied [23,24,25,26,27]. Starch characteristics like granule size, and particularly the DP of AML and AMP, really need to be studied using a dedicated tool for polymer characterization in regard to the current genetic and environmental factors involved in wheat culture.

Considering the current and projected environmental impacts (i.e., climate change with increasing heat and/or water stresses in particular), it is essential to better understand these phenomena to implement new breeding strategies for sustainable quality. In this study, 14 winter wheat cultivars were grown under field conditions in locations widely scattered across France and Europe, and flours were subjected to a comprehensive analysis of wheat starch traits. The main objective was to assess the effects of genetic (G), environment (E) and G × E interaction on the starch parameters. In addition, the present study offers a comprehensive discussion on variations of starch biosynthesis.

## 2. Materials and Methods

### 2.1. Multilocal Trials and Plant Material

**Figure 1:**
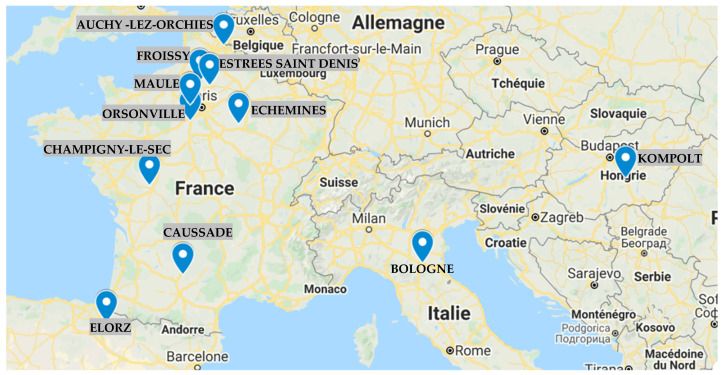
Locations where the experimental trials were carried out two consecutive growing seasons (2015 and 2016; Caussade has been excluded from the study).

In the frame of the collaborative research project established between Arvalis, UniLaSalle and private European plant breeding companies working in France, multilocal trials were carried out during two different growing seasons (2015 and 2016) (Program “IGE: Understanding the effect of genetic–environmental interactions on breadmaking value”). Fourteen winter wheat cultivars were grown in ten locations in the European areas, providing a very large contrast of growing environments (Figure 1).

Wheat cultivars were chosen as function of:

- Stability of their known bread-making quality. Some of them are considered stable and some varieties are recognized to be less stable.
- The allelic form at the GluD1 locus considering the weight of this gene on the final rheological quality.
- Earliness to heading stage which may allow adaptation to very wide geographical locations exhibiting different pedo-climatic conditions.

Ten experimental sites were identified by the breeders based on the quality of the results regularly observed at these locations, with the aim of maximizing the sources of variability in the expression of breadmaking quality.

Experimental trials were conducted in the field, including randomized plots with three replicates. Conventional agronomic practices, with mineral and fungicide treatments, were used to achieve optimal grain yield without any water irrigation.

Three kinds of locations were chosen:

- Classically adapted sites (“typical” or “standard” sites in France) consisted of locations with a low frequency of abiotic stress where regularly stable breadmaking quality is obtained in the control cultivars.
- Classically invalidated sites (“atypical” sites in France) with episodes of heat and hydric stress leading to regularly variable breadmaking values in the control cultivars.
- Sites with restrictive climatic conditions, particularly end-of-cycle temperatures from European networks (EU sites).

### 2.2. Flour Starch Extraction

Starch was extracted using the method developed previously with some technical modifications [28]. Wheat grains were ground down to flour using RETSCH laboratory ball mills (Verder Scientific, Eragny-sur-Oise, France). In order to purify starch granules from the rest of the constituents (mainly proteins), 3 g of whole flour was washed twice for about 30 min and three times for 60 min with 15 mL of washing solution (55 mM Tris-HCl, pH = 6.8, 2.3% (*w*/*v*) SDS, 1% (*w*/*v*) dithiothreitol (DTT), 10% (*v*/*v*) glycerol) at room temperature. At each washing step, starch granules were disrupted with sonication for 20 s at a power setting of 20% using a stepped microtip probe (6 mm diameter) (Sonics Materials, Bioblock Scientific, model 75038, Fisher Scientific SAS, Illkirch, France). Granules were then washed three times for 5 to 10 min with cold water, once with cold acetone and finally air-dried at room temperature overnight. Each purification step was followed by centrifugation at 3500× *g* for 5 min. The extractions were conducted in triplicate.

### 2.3. Flour Total Starch Content

The total starch content of whole flour was calculated using a Total Starch Amyloglucosidase/α-Amylase Assay Kit (Megazyme International Ireland Ltd., Bray, Ireland). Wheat flours (100 mg) were weighed, in triplicate, into a 20 mL Pyrex^®^ glass tube. The samples were wetted with 0.2 mL of an 80% (*v*/*v*) aqueous ethanol solution for dispersion and stirred on a vortex mixer. Then, 3 µL of thermostable α-Amylase prepared in 3-Morpholinopropanesulfonic acid (MOPS) solution buffer was added to the slurry and thoroughly and vigorously stirred on a vortex mixer. To start enzymatic digestion, samples were incubated at 100 °C for 6 min with stirring every 2 min. The tubes were then placed in a water bath at 50 °C and 4 mL of sodium acetate solution (200 mM, pH = 4.5) was added. Amyloglucosidase preparation (0.1 mL) was introduced and samples were stirred on a vortex mixer and incubated for 30 min at 50 °C. After incubation, the samples were transferred to 50 mL tubes and the volume was then adjusted with 0.7 mL of distilled water. The samples were mixed vigorously and centrifuged at 3000 rpm for 10 min at 20 °C. The diluted solutions (1 mL) were transferred in triplicate to Pyrex^®^ glass tubes. A solution (3 mL) of glucose oxidase peroxidase 4-aminoantipyrine (GOPOD) reagent was added to tubes and incubated for 20 min at 50 °C. Glucose controls consisted of 0.1 mL of glucose standard solution, while reagent Blank Solutions consisted of 0.1 mL of distilled water. The absorbance was read against the reagent blank at 510 nm for each tube including that containing the glucose control. The starch content was expressed as a percentage of flour dry weight (DW).

### 2.4. Distribution of Starch Granules

The size distribution of the purified starch granules was measured with a Mastersizer 2000 laser particle size analyzer (Malvern Panalytica, Palaiseau, France) following the protocol previously reported [29]. Each starch sample was analyzed three times.

### 2.5. Characterization of the Molecular Weight Distribution (MWD) of Grain Storage Starch Polymers by Asymmetric Flow Field-Flow Fractionation (A4F)

Starch was solubilized according to the method established previously [30]. About 10 mg of starch granules was dispersed, in triplicate, with 1 mL of a dimethylsulfoxide (DMSO)/water solution (95%) (*v*/*v*) and heated for 60 min at 110 °C. Gelatinized starch was precipitated using 5 mL of absolute ethanol and then centrifuged at 20,000× *g* for 20 min at 20 °C. Supernatants were discarded, while pellets were kept and mixed with 4 mL of NaOH solution (20 mM) and transferred into 10 mL pressurized vessels (CEM, Saclay, France). Solubilization was conducted using microwave heating at variable power (Discover, CEM, Saclay, France) for 8 min at 135 °C. Solubilized starchy polymers were then filtered through 5 µm cellulose nitrate syringe filters (Pall, Saint-Germain-en-Laye, France) and 100 µL was injected into the A4F system. Pullulan standard polymers (110 to 800 kDa) were used for initial operational checking of the whole Eclipse™ A4F system. A4F analysis was accomplished using an Eclipse3 F System (Wyatt Technology, Santa Barbara, CA, USA) combined with a multi-angle light scattering (MALS) detector (Dawn^®^ multi-angle Heleos™, Wyatt Technology, Santa Barbara, CA, USA) and an Optilab^®^ T-rEX™ refractive index (RI) detector, (Wyatt Technology, Santa Barbara, CA, USA). The light scattering detector was calibrated using toluene as a standard. Constant calibration of the RI detector was calculated using sodium chloride at different concentrations and the temperature was set at 35 °C. The short separation channel (195 mm in length) had a trapezoidal geometry. The thickness of the spacer was 0.35 mm. The accumulation wall was constituted of regenerated cellulose ultrafiltration membrane with a cut-off of 10 kDa (Superon). An Agilent HPLC 1200 Series (Agilent Technologies, Waldbronn, Germany) was used in tandem with the A4F system. The eluent was ultra-pure deionized water with 0.02% NaN_3_ (*w*/*v*) added as a preservative. The mobile phase was degassed and filtered through a 0.1 µm inline filter (Pall, Saint-Germain-en-Laye, France) before entering the A4F channel.

Separation of AML and AMP was achieved following the protocol reported in our previous work [30]. AML and AMP contents is expressed as a percentage of starch dry weight.

The polymer characteristics of AML and AMP measured using A4F, were: weight-average molar mass Mw (expressed in g/mol); number-average molar mass Mn (in g/mol); polydispersity Mw/Mn; and gyration radius R (in nm).

### 2.6. Statistics

All statistical analyses (i.e., descriptive statistics, simple regression, Pearson correlation and ANOVA with a general linear model, GLM) were performed using Statgraphics^®^ software. Statistical significance was accepted at *p* < 0.05, *p* < 0.01, *p* < 0.001 and *p* < 0.0001.

## 3. Results and Discussion

### 3.1. Starch Characteristics Exhibited Large Phenotypic Variations

Overall means of starch content, starch granule distribution and molecular features of the 14 wheat cultivars grown in the 20 growing conditions (Year × Location) are shown in Table 1.

Starch content for all wheat samples varied from 54% to 69.48% with a mean value of 61.10%, showing a low coefficient of variation (CV) of about 3.66%. The granule content distribution showed higher levels for A-type granules with a mean of 75%, followed by B-type granules, with an overall mean of 21%, then C-type granules with an overall mean of 4%. Percentage volume of A granules which represents the highest fraction, showed the lowest CV (~4%) amongst total granule distribution. This variability could be related to the low variability observed for total starch content. The widest range of granule distribution was observed for B-type granules with a CV of ~14% followed by C-type granules with CV of ~11.76 (Table 1). These results evidenced that B-type and C-type granules had higher amounts of exploitable genetic variability than the starch content and volume of A-type granules.

Separation of AML and AMP showed mass fraction proportions, respectively, of 30% and 70% (Table 1). These relative fractions are in accordance with previous statements since AML/AMP ratio is about 1:3 in non-mutant endosperm starch [31,32]. Even though AML represents the smallest fraction, it showed more variability in comparison with the AMP fraction showing twice the CV value (6.7% vs. 3.34%). It is important to elucidate the factors involved in this variation since the amount of AM present in granules has been found to significantly affect the physico-chemical and functional properties of starch [33].

Characterization of the MWD of grain storage starch polymers using A4F was here performed for the first time in a research program studying genotypic and environmental effects on bread wheat. Mw, Mn, Mw/Mn and R for AML and AMP and Mw/Mn means and CV are shown in Table 1. AML Mw ranged from 0.1 × 10^6^ to 1.4 × 10^6^ g/mol with a mean value of 0.4 × 10^6^ g/mol. The MWD has not been widely examined for wheat starch; however, it is known that the Mw of AML varied from 10^5^ to 10^6^ g/mol [34]. AMP Mw ranged from 10.4 × 10^6^ to 58.4 × 10^6^ g/mol, with a mean of 23.4 × 10^6^ g/mol. Molar masses were reasonably consistent with those reported for AML and AMP from several cereals including barley, triticale and wheat [35]. In the literature, AMP Mw varies from 10^7^ to 10^9^ g/mol [30,36,37,38]. However, the masses obtained in our study are lower than those of these last reports and differences could be attributed to the starch solubilization process and/or the source of starch material. In our previous study on five different maize hybrids, the observed Mw ranged from about 2 × 10^5^ to 4 × 10^5^ g/mol for AML and from 1 × 10^8^ to 4 × 10^8^ g/mol for AMP [30].

AML and AMP were moderately to highly dispersed since AML showed an overall mean Mw/Mn value of 1.65 while AMP exhibited a value of 4.41. As a result, starch was extremely dispersed, showing an overall mean value of 24.68. The results indicate very strong heterogeneity of the lengths of the starchy chains formed during grain filling.

Values of the AMP gyration radius, Rw, ranged from 53.2 to 141.9 nm. It showed an overall mean of 95.61 nm, twice that of AML (48.92 nm). Values agreed reasonably with those found for AML and AMP in different species [35]. The higher Mw and lower Rw of AMP suggest that AMP chains were compactly packed and highly branched.

On the other hand, all molecular attributes showed wide dispersity among the 14 cultivars and the 20 growing conditions, with CV values ranging from 13.17% to 34.04% for AMP gyration radius and AML Mn, respectively. The highest CVs were obtained for the molecular weights of the AML and AMP chains. These results, mainly those related to molar masses, imply that the molar masses had a higher level of useful genetic variability among all starch attributes studied. Greater CVs for starch attributes could indicate greater potential for favorable advances in wheat breeding.

### 3.2. Genetic Impacts on Starch Characteristics

The relative contribution of cultivar (G), environment and G × E interaction to the variation observed in starch, starch granule distribution and the molecular properties of starch polymers is reported in Table 2.

Genetic was a highly significant (*p* < 0.0001) source of variation for starch content, starch granule distribution and for all molecular features of its components (Table 2). The relative magnitude of the genotypic contribution to the distribution of different granule types (A, B and C) was considerably larger (54–74%) than its contribution to the molecular features of AMP (9–22%) and AML (5–10%) features. These significant impacts showed, despite the similar earliness, the presence of genetic differences between the 14 cultivars for all traits. Larger environmental differences between the 20 growing conditions made it possible to detect genotypic differences even some small ones. However, it is often demonstrated that the genetic factor has a significant impact on the amount of starch and the percentage of AML [38]. Our results also corroborated numerous studies showing a strong genetic influence in the establishment of the size, morphology and relative percentage of granules [39,40,41]. This control is related to the fact that wheat endosperm starch is synthesized by an enzymatic arsenal, implying a high number of genes [42]. Among these enzymes, adenylytransferase (AGP), soluble starch synthase (SSS), GBSS, SBE and the DBE family are the most important enzyme families [43,44,45]. The identification and impact of certain enzymes involved in the molar mass and size of starch polymers (AML and AMP) remain to be elucidated.

The maximum, minimum and CV% attributed to G, E and G × E interaction for starch content, starch granule distribution and MWD of starch polymers are reported in Table 3. In addition, the genetic CV calculation showed narrow ranges in starch content, percentage of A-type granules, and AML and AMP mass fractions. The most important variations were found for polymer molecular characteristics. Cultivars varied slightly to moderately in their AMP mass fraction, starch content, percentage of A-type granules and AM mass fraction showing CV values of 3.66%, 3.92%, 5.09% and 6.9%, respectively. The differences in the averages for these traits remained relatively small. The lower CVs suggest that cultivars tended towards being more stable or less variable across growing conditions for these traits. This stability could be due to the varieties having a similar earliness characteristic. In addition, in our cases, these traits may not explain the differences in breadmaking quality that exist between the selected cultivars.

B- and C-type Starch granules showed a higher CV in comparison with A-type granules. B-type granules exhibited a CV value of 15.45% while C-type granules showed a CV value of 13.50%. This is in accordance with reported results [46]. Wide variability of starch granules is found in the literature, attributed to several factors including the wheat cultivar [47]. It should be noted that properties such as gelatinization, swelling and other technological characteristics have been found to be affected by the ratio of starch granules [48]. In addition, granules differ in their molecular composition, mainly the AML/AMP ratio and the branch chain-length distribution of AMP [46]. They can present differences in chain-length distribution of AML and AMP. Thus, variations in these traits could be the source of variations in technological qualities between wheat cultivars.

Large variations were determined for the molecular features of starch polymers (Table 3). CV values ranged from 8.51% for AML dispersity (Mw/Mn) to 37.40% for AMP molecular mass (Mn). Higher CV values suggest that the cultivars were less stable or were subject to variation in growth conditions. The largest variations were found for the molar masses of AML and AMP. Despite genotypic stability in the amount of starch and the AML/AMP ratio, the starch deposited during the grain filling phase showed differences in the elongation, branching and dispersity of the glucan chains. These variations of molecular features could be related to the branch chain-length distribution of glucan chains, and together led to variations in granule composition and morphology.

These data indicate that AML and AMP molecules are likely genetically controlled during their biosynthesis, as previously reported [49]. These variations between cultivars can be attributed to differences of gene expression levels and/or variations in enzyme activity.

Since cultivars showed large variations in these traits and these variations were genetically controlled, the traits studied here can be considered in wheat breeding programs.

### 3.3. Environment Effects on Starch Characteristics

Environment CV (CV_E_) calculations showed very wide ranges in all parameter means studied except for starch whole flour content, percentage of A-type granules and percentage of AMP mass fractions (Table 3). The most important variations were found for polymer molecular features. Their CV values ranged from 9.08% (AML mass fraction) to 47.36% (AML Mw). In addition, the molar mass of AML and AMP with starch dispersity parameter exhibited the largest variations (more than 25%).

As expected, the ANOVA components showed a significant main effect of the environment factor (*p* < 0.0001) for starch content, granule distribution, AML% and AMP% (Table 2). The effect of the environment on these three quantitative parameters has been widely documented, particularly for starch content. All the studies agree that starch content is strongly influenced by environmental factors. Depending on the study, the factors responsible for the variations in starch content seem to be growing season temperature, rainfall patterns, soil moisture, irrigation, growing area, sustained changes of the climate, or episodic stresses [38,50,51,52,53,54,55]. High temperatures, between 30 and 40 °C, decrease the wheat starch concentration by 2% to 33% [50,56]. A temperature higher than 30 °C during the first days after anthesis has the most effect on the accumulation of starch [50]. The phenomenon is accentuated when the temperature rises above 35 °C.

Contrary to the previous parameter, very few studies have addressed the environmental effects on the distribution of starch granules and the percentage of AML [51,57]. Our results reinforce these studies, indicating that the environmental conditions are the main factors explaining the increase in variability. Heat stress or elevated temperature during growth, mainly the grain filling period, have been shown to alter starch granule distribution, shape and structure [31,50,58]. Temperature variation decreases characteristics of granules such as their size and quantity in the wheat endosperm [31,59,60,61,62]. The proportion of A-type granules increases at the expense of the size and number of the small granules (B and C). The size of A-type wheat granules is most and drastically impacted when heat stress occurs close to the anthesis period [50]. Growing conditions with temperatures above 30 °C during the first days of grain filling produce wheat seeds with a high proportion of A-type starch granules [60]. In some cases, temperature, depending on the extent of severity and duration of heat stress, may result in cracks and pitting on the surface of the granules [50]. Wheat grown at 40 °C for 72 h showed morphological deformations and cracks, in addition to the effects on A-type granules [50].

Starch granule size distribution in wheat endosperm may also be affected by water deficit. Granule proportions vary only under a long period of water stress [63]. Water deficit clearly reduces the size of all starch granule types [63,64,65]. It has been reported that water-saving irrigation and rainfed experiments increase the volume proportion of B-type granules and decrease that of A-type granules compared to experiment with normal irrigation [39]. The content of AML and AMP can be influenced by water deficit [38,66]. Wheat experimented on under drought conditions showed a lower AML content [64,65].

The AML content may change as a function of ambient temperature conditions during the grain filling period. High temperature has been shown to increase wheat AML content and decrease the AMP/AML ratio [31,32,50,58,60,67,68,69]. It has been shown that AML content increases slightly due to terminal growth heat-shock [58], while it has been reported that high temperature during the first days after anthesis (6–8 DAA) has the greatest impact and raises the AML content [50]. In addition, the increases of AML content were related to temperatures above 30 °C during the first 14 DAA [60]. When wheat plants were kept in a greenhouse at 40 °C for 72 h, most of them accumulated starch with a low level of AML [70].

For the first time, the molecular features of starch polymers were tested for the influence of environment. Without exception, the results showed significant environmental impacts (*p* < 0.0001) for all features (Table 2). Large variations in molar mass parameters (Mn and Mw) were found. The Mn of AML ranged from 0.16 × 10^6^ g/mol at Orsenville (France) in the 2015 crop season, to 0.52 × 10^6^ g/mol at Champigny (France) in the 2015 crop season. The same results were obtained for Mw which varied between 0.25 × 10^6^ g/mol and 0.69 × 10^6^ g/mol. It should be noted that the latter is one of the atypical locations. In addition, the Mn of AMP oscillated from 3.673 × 10^6^ to 8.15 × 10^6^ g/mol at Estrées Saint Denis (France) and Navarre (Spain), respectively, in the 2015 crop season. The Mw ranged from 18.64 × 10^6^ g/mol in an atypical area, Échemines (France) in the 2015 crop season, to 30.51 × 10^6^ g/mol at Navarre (Spain) in the same crop season. To verify our choice of locations used in our study and to detect possible effects exerted by groups of locations, we processed the data according to the characteristics of the locations. Thus, we have formed three groups of places as indicated in the materials and methods section. The means and the results of the Duncan analysis are shown in Table 4.

The results showed that there were significant differences between location groups for all parameters except for the percentage of AML and AMP. We noted that “typical” locations in France and European areas were often distinguished from the “atypical” group in France. This really indicated significant differences in growing conditions (abiotic stress). Typical locations in France showed higher values for molecular features (Mn, Mw and Rw) for AML and AMP than those found in atypical locations. In-depth analyses will be carried out to understand and determine the exact source of these differences. We will be able to determine the weight of each factor (temperature, precipitation, temperature at the end of the cycle, etc.) using advanced statistical analyses (PLS for example).

### 3.4. Genetic by Environment Interaction

In the current study, the results show that environment was the major source of variation for biosynthesis of starch and its components (Table 2 and Table 3).

CV_E_/CV_G_ ratios clearly show the relative importance of the growing conditions on all molecular parameters except for starch content and starch granules (Table 3). For A- and B-type granules, the genotypic variations were slightly less important than the environmental variations. while C-type granules were equally controlled by both factors.

For the rest of the parameters, i.e., the molecular aspects, the of environmental factors had a greater influence than the cultivar differences. The most important variation was found for starch molecule dispersity as demonstrated by a ratio of around 2, whereas the lowest ratio was obtained for Mw of AMP where environmental variation was only about 1.1 times greater than the respective genotypic variation. In contrast, the growing environment variations for Mn and Mw of AML were 1.89 and 1.54-fold greater than genotypic CV.

Genetic × environment interactions were, without exception, significant for all the parameters studied at the *p* < 0.0001 level (Table 2). The relative contribution of G × E to total variance was substantially smaller than that of genetic or environment, it ranged from 2.09% to 17.76% for starch dispersity and Mw of AMP, respectively. The most important contributions were found for the molar masses and gyration radius of AMP.

The relative magnitude of the environmental contribution to variance in starch traits was much greater than the contribution of either genetic or G × E interaction (Table 2). An exception was noticed for granules where the cultivar had a larger contribution of variance than environment. The relative contribution of the growing environment ranged from 21.77% to more than 95% while those of genetic and G × E interaction varied between 2.71% and 73.45%, and 2.09% and 17.76%, respectively. As far as molecular parameters are concerned, the relative contribution of the environment completely overshadowed the contribution of other factors. The mean contribution of environment for these molecular properties was about 80%, it was about 9.5-fold more important than genetic factor and 10 times more than G × E interaction. In other words, the AML/AMP ratio, dispersity, molar masses and radii of gyration of AML and AMP were very sensitive to the effects of environmental conditions during plant growth.

Overall, this shows that environmental conditions act on starch metabolism pathways. However, variations observed in all parameters between genetics grown in the same environmental conditions indicate that the genetic factor may play a determinant role in controlling these variations.

### 3.5. Starch Biosynthesis

Starch biosynthesis and accumulation is synchronized with the filling and development of the wheat grain. It starts early during the first days after anthesis (4–10 DAA), corresponding to endosperm differentiation or cell division phases [71]. Starch deposition level increases gradually during grain filling and stops during the desiccation phase [72,73]. Starch biosynthesis, the starting point of which is glucose and/or fructose and/or sucrose, is genetically controlled implying numerous genes and related enzymes.

Reported RNA-Seq results highlighted 166 homologs of starch biosynthesis-related genes expressed during the development of wheat grain endosperm [45]; 74 selected from 17 gene families and expressed at particularly high levels during 8–20 DAA are related to reserve starch deposition. In addition, co-expression analysis of potential regulators has shown that 425 transcription factors (TFs) are involved in regulating the expression of starch biosynthesis-related genes in hexaploidy wheat [45]. The first step consists of converting glucose-1-phosphate to ADP-glucose with AGP. SSs then catalyze the interaction between ADP-glucose and the non-reducing terminus of the growing chain of glucose residues to form α-1,4-glycosidic linkages. There are five groups of these enzymes. The SSS family catalyzes AMP elongation while GBSS alone is responsible for AML biosynthesis [13]. SBE create α-1,6-glycosidic bonds between chains to form branched AMP molecules [44]. SBE are responsible for the hydrolysis of α-1,4-glycosidic bonds and transfer of the cleaved polymer to an acceptor chain through an α-1,6-glucosidic branching point [74]. Depending on enzymatic function, SBE are formed of at least two families [74,75,76]. Another kind of genetic control takes place via the DBEs family of enzymes which catalyze the hydrolysis of α-1,4-glycosidic bonds within chains. Their activity regularizes the branching and crystallinity of AMP molecules [77,78]. It is clear that genetic control is achieved via a number of very important enzymes intervening in carbonaceous metabolism, more particularly in the starch synthesis pathways. Depending on the sensitivity of these enzymes and/or the genetic and/or biochemical factors controlling their expression and activity, environmental conditions can affect the starch qualitatively and quantitatively. However, growing conditions can alter starch deposition by acting on these enzymes involved in substrate availability, chain elongation, branching, debranching, crystallinity control and the formation of granules [79].

Any punctual and severe environmental event during the starch deposition phases would have variable effects depending on the stages of wheat grain development and on the sensitivity of starch biosynthesis-related genes and their regulators. Thus, AGP, SS, GBSS and SBE are reported to be the most sensitive to abiotic stress such as high temperature and drought [31,43,80,81]. Under heat stress, transcriptional analysis of starch biosynthesis genes shows variations of gene expression in wheat and rice endosperm [31,80]. These variations are always accompanied by identical changes in overall enzyme activity [82,83].

Variations in the transcripts, translation and enzyme activity could explain the genotypic and environmental variations observed in our study. Changes in SS activity could explain variations of starch content since SSs have been shown to be the most sensitive to drought and largely explain the starch content in grain wheat [84]. In wheat, SSs can be inactive at very high temperature and then accumulation of starch can stop [43]. Reduced activity of SSs at high temperature such as 30 °C does not affect starch deposition but changes its composition. Since SSs are involved in the biosynthesis of AMP. Genetic and environmental variations in granules and the molecular features of AMP could be explained by the activity of SSs and related genes.

In addition, activity of AGP could also explain variations in starch content as it is involved in the most limiting step of starch biosynthesis by providing glucose molecules in the form of ADG-glucose. AGP activity declines drastically under water stress and leads to premature cessation of starch accumulation [84,85]. It has also been found to be the most sensitive to elevated temperature [43,86].

GBSS is considered to be less sensitive to elevated temperature while it is significantly affected by drought [82,84,87]. As a consequence, variation of the AML content, AML to AMP ratio, granule distribution and molecular features of AM could be attributed to alteration of GBSS activity. This is supported by lower grain AML content in rice under water deficit being related to lower GBSS expression attributed to transcriptional regulation of GBSS [88].

SBE activity seems to be sensitive to heat stress in maize and rice [83,89]. The variation in SBE activity could then explain the variation in the length of the glucan chain and, consequently, the variations in the molar masses of the starch molecules and also all the other molecular and granular characteristics.

## 4. Conclusions

For all of the French bread wheat parameters tested, environmental growing conditions were considerably more important than variations associated with genetic factor, being excluded to starch content and granule distribution. This obviously reveals the importance of growing conditions on starch components and related variables. As expected, the relative contribution of the G × E interaction to total variation was substantially lower than that of either genetic or environment.

Our results point clearly to the presence of genetic mechanisms controlling starch polymerization and structuration (i.e., molar masses, gyration radius, granule distribution, etc.) that can be considered by breeders in future wheat breeding programs, since some variations of these traits have already been related to technological differences between cultivars. However, genetic and enzymatic mechanisms were influenced by growing environment. In order to maintain a product of uniform and expected quality, the challenge for the common wheat industry is to manage as much as possible the impacts of the changing growing environment on wheat quality by stabilizing not only protein-related parameters, but also starch-related characteristics.

## Figures and Tables

**Table 1:** Mean ^1^ starch content, starch granules distribution and starch molecular characteristics of 14 wheat cultivars grown in 20 growing conditions.

Traits	Mean ^1^	Min	Max	SD ^2^	CV ^3^
Starch content (%)	61.10	54.00	69.48	2.23	3.66
A granules (%)	74.28	54.59	90.00	3.29	4.43
B granules (%)	21.14	10.86	37.65	2.97	14.08
C granules (%)	4.17	2.59	7.75	0.49	11.76
AML					
Mass fraction (%)	30	17	41	2	6.79
Mn (10^6^ g/mol)	0.24	0.09	0.76	80	34.04
Mw (10^6^ g/mol)	0.38	0.14	1.38	106	28.37
Mw/Mn	1.65	1.25	3.69	0.17	10.07
R (nm)	48.92	29.00	104.10	7.33	14.99
AMP					
Mass fraction (%)	69	58	82	2	3.34
Mn (10^6^ g/mol)	5.66	1.40	28.08	1.871	33.06
Mw (10^6^ g/mol)	23.46	10.44	58.43	4.795	20.44
Mw/Mn	4.41	2.00	9.34	0.66	14.96
Rw (nm)	95.61	53.20	141.90	12.60	13.17
Total Starch fraction					
Mw/Mn	24.68	9.72	47.49	5.79	23.46

^1^ Means of three replicates and 20 growing conditions. ^2^ SD: Standard deviation. ^3^ CV: Coefficient of variation (%). AML: amylose. AMP: amylopectin.

**Table 2:** Contribution of variance components to variation (percentage of total estimate) for environment (E), genetic (G) and G × E interaction effects for starch composition and molecular features of its components.

Traits	Variance Component			
	E (%)	G (%)	E × G (%)	Residual (%)
Starch content	55.86 ***	37.28 ***	4.22 ***	2.64
A granules	21.77 ***	73.45 ***	3.62 ***	1.15
B granules	29.75 ***	66.67 ***	2.96 ***	0.62
C granules	39.05 ***	53.81 ***	6.13 ***	1.00
AML				
Mass fraction	82.89 ***	9.67 ***	6.35 ***	1.09
Mn	92.06 ***	4.75 ***	3.10 ***	0.09
Mw	86.20 ***	8.15 ***	5.42 ***	0.22
Mw/Mn	86.47 ***	7.03 ***	5.94 ***	0.56
Rw	85.76 ***	7.33 ***	6.46 ***	0.45
AMP				
Mass fraction	78.96 ***	12.16 ***	5.72 ***	3.16
Mn	60.24 ***	22.35 ***	17.11 ***	0.29
Mw	72.40 ***	9.07 ***	17.76 ***	0.76
Mw/Mn	78.96 ***	11.75 ***	8.78 ***	0.51
Rw	77.16 ***	10.24 ***	11.63 ***	0.97
Total Starch fraction				
Mw/Mn	95.02 ***	2.71 ***	2.10 ***	0.18

*** Significance at 0.0001 probability level.

**Table 3:** Coefficients of variation (CV) due to environmental (E) and genetic (G) effects for starch composition and molecular features of its components.

Traits	Environment ^1^ (%)			Genetic ^2^ (%)			CV_E_/CV_G_
	Min	Max	CV_E_	Min G	Max G	CV_G_	
Starch content	58.63	63.59	4.31	58.88	62.64	3.92	1.09
A granules	71.76	78.92	4.56	69.04	79.65	5.09	0.89
B granules	15.66	23.66	14.21	16.61	26.44	15.45	0.91
C granules	3.72	5.03	13.46	3.61	4.71	13.50	0.99
AML							
Mass fraction	24	33	9.08	29	31	6.90	1.31
Mn	0.16	0.52	47.36	0.22	0.27	24.97	1.89
Mw	0.25	0.70	38.81	0.33	0.44	25.17	1.54
Mw/Mn	1.36	1.86	12.86	1.60	1.73	8.51	1.51
Rw	40.25	65.39	19.81	45.87	51.95	14.04	1.41
AMP							
Mass fraction	66	75	4.50	68	70	3.66	1.22
Mn	3.67	8.16	43.31	4.67	7.34	37.40	1.15
Mw	18.65	30.52	25.60	21.91	24.96	23.21	1.10
Mw/Mn	3.62	5.95	19.85	4.19	4.92	15.24	1.30
Rw	78.40	111.48	16.20	91.61	99.58	13.81	1.17
Total Starch fraction							
Mw/Mn	12.06	33.08	28.88	22.98	26.00	14.63	1.97

^1^Mean CV of 3 repetitions and 20 environments (growing locations). ^2^ Mean CV of 14 cultivars.

**Table 4:** Overall means of starch content, starch granule distribution and molecular features of 14 wheat cultivars in relation to growing location groups.

Traits	France “Typical” Locations	France “Atypical” Locations	Europe Areas
Starch (%)	61.26 ^b,1^	61.31 ^b^	60.66 ^a^
A granules (%)	74.14 ^b^	73.34 ^a^	75.66 ^c^
B granules (%)	21.01 ^b^	22.25 ^c^	19.78 ^a^
C granules (%)	4.26 ^b^	4.10 ^a^	4.18 ^a,b^
AML			
Mass fraction (%)	30 ^a^	30 ^a^	30 ^a^
Mn (10^6^ g/mol)	0.26 ^b^	0.22 ^a^	0.23 ^a^
Mw (10^6^ g/mol)	0.40 ^b^	0.35 ^a^	0.38 ^b^
Mw/Mn	1.63 ^a^	1.67 ^b^	1.64 ^a,b^
Rw (nm)	50.17 ^b^	48.11 ^a^	48.73 ^a,b^
AMP			
Mass fraction (%)	69 ^a^	69 ^a^	69 ^a^
Mn (10^6^ g/mol)	5.62 ^a^	5.26 ^a^	6.23 ^b^
Mw (10^6^ g/mol)	23.61 ^b^	22.28 ^a^	24.86 ^c^
Mw/Mn	4.28 ^a^	4.67 ^b^	4.20 ^a^
Rw (nm)	96.36 ^b^	92.71 ^a^	98.71 ^b^
Total Starch fraction			
Mw/Mn	23.06 ^a^	25.93 ^c^	24.62 ^b^

^1^ Means with the same letters do not differ significantly by Duncan test at *p* = 0.05.

## Data Availability

The data presented in this study are available on request from the corresponding author. The data are not publicly available due to the fact that we did not get the authorization from the plant breeders. This is why the 14 varieties have been coded and treated confidentially.
